# The serum level of 25-OH vitamin D and Th1 cytokine pattern in HIV infection versus hepatitis C virus infection and hepatitis B virus infection

**DOI:** 10.1186/1758-2652-13-S4-P102

**Published:** 2010-11-08

**Authors:** SA Iacob, D Banica, E Panaitescu, M Cojocaru, D Iacob

**Affiliations:** 1 Carol Davila University of Medicine, Infectious Diseases, Bucharest, Romania; 2 Carol Davila University of Medicine, Immunology, Bucharest, Romania; 3 Carol Davila University of Medicine, Biostatistics, Bucharest, Romania; 4 Titu Maiorescu University, Immunology, Bucharest, Romania; 5 Carol Davila University of Medicine, Student, Bucharest, Romania

## Purpose of the study

To assess the plasma level of 25- hydroxy vitamin D (25-OHD) and its correlations with the immune status in patients infected with Human Immunodeficiency Virus Infection (HIV), Hepatitis C Virus Infection (HCV) and Hepatitis B virus Infection (HBV).

## Methods

The study was performed on 54 patients admitted to Matei Bals Institute Bucharest, between January 2010 - July 2010, out of which 30 women and 24 men aged 21-64 years. 14 patients were diagnosed with HIV stage C3(CDC criteria), 11 with HBV and 14 with HCV infection, while 15 were healthy controls. We assessed the plasma level of 25-OHD(nmol/L, Elisa kit, Immunodiagnostic Systems), IFN γ, IL2, IL12(pg/mL, Max Discovery Elisa kit), phosphorus and calcium level, the CD4, CD8 cell count and the CD4/CD8 index. Kruskal-Wallis and ANOVA comparative tests were further used to determine the p value.

## Summary of results

HIV patients displayed the lowest plasma concentration of 25-OHD(27.13 nmol/L) despite no statistically significant difference towards controls. No correlation was found between the 25-OHD level and any of the studied parameters. The serum level of IFN γ in HIV patients was similar with the concentration found in controls; a significant positive correlation was found with the CD8 count(p=0.01) and the CD4/CD8 index(p=0.008). The IL2 concentration was decreased and it was correlated with the level of IL12(p=0.000) and the CD4 count(p=0.02). The low level of 25-OHD in HCV patients (28.97 nmol/L), was correlated with the CD4 count(p=0.008) and the CD4/CD8 index (p=0.003); IFN γ displayed a considerable increased level while the concentrations of IL2 and IL12 were within the normal range. 25-OHD was also decreased in HBV patients and its serum level was correlated with the CD4 count(p=0.01), as well as the CD8 count(p=0.06). The serum concentration of IFN γ was increased compared with controls.

## Conclusions

The 25-OHD levels were lower in HIV, HCV and HBV patients. Nonetheless a similar insufficiency was also noted in healthy controls. No correlation was further determined between the level of 25-OHD and the immune status in any group of patients. The Th1 cytokine pattern was different in the HIV infection compared with HCV and HBV infection. IL2 and IL12 serum level was significantly decreased in HIV infection; IFN γ exhibits a significant increased serum level in HCV infection. This work was supported by CNCSIS-UEFISCU Grants PNII IDEI code 2508/2008 (project no. 1165)

